# Model-Based Product Temperature and Endpoint Determination in Primary Drying of Lyophilization Processes

**DOI:** 10.3390/pharmaceutics14040809

**Published:** 2022-04-07

**Authors:** Alex Juckers, Petra Knerr, Frank Harms, Jochen Strube

**Affiliations:** 1 Institute for Separation and Process Technology, Clausthal University of Technology, 38678 Clausthal-Zellerfeld, Germany; juckers@itv.tu-clausthal.de; 2 Martin Christ Gefriertrocknungsanlagen GmbH, 37520 Osterode am Harz, Germany; p.knerr@martinchrist.de (P.K.); f.harms@martinchrist.de (F.H.)

**Keywords:** lyophilization, process modeling, quality by design, process design and optimization, primary drying endpoint determination, product temperature profile

## Abstract

Lyophilization process design still relies mainly on empirical studies with high experimental loads. In the regulatory demanded Quality by Design approach, process modeling is a key aspect. It allows process design, optimization and process control to ensure a safe process and product quality. A modeling approach is outlined that is able to predict the primary drying endpoint and temperature profile of distinct vials. Model parameters are determined by a reproducible determination concept. Simulated results are validated with a fractional factorial Design of Experiments (DoE) in pilot scale. The model shows higher accuracy and precision than the experiments and similar parameter interactions for both the endpoint and temperature determination. This approach can now be used to explore the primary design space in lyophilization process design. This paper proposes a distinct method for endpoint determination and product temperature prediction by a modeling approach based on Velardi et al. combined with a distinct model parameter determination according to Wegiel et al. and Tang et al.

## 1. Introduction

The control of the product temperature and drying endpoint in the primary drying phase of lyophilization processes is of utmost importance in order to ensure a safe process where the product meets the quality attributes while maintaining the highest possible productivity [1,2,3]. Generally, the lyophilization process can be divided into three consecutive steps: freezing, primary drying and secondary drying. During the freezing step, the product solution is cooled down in order to convert the liquid water into ice while the solutes transform into a crystalline or amorphous solid. The final system temperature is dependent on system characteristics. A crystalline solute must be cooled down under the eutectic melt temperature while an amorphous system must be cooled down under the glass transition temperature of the maximally freeze-concentrated solute to ensure crystallization or vitrification of the solutes [4,5,6]. The formed ice is then removed by sublimation during primary drying. Here, the chamber pressure is lowered to a value under the triple point of water in order to generate a driving force for sublimation. The necessary heat for the endothermic sublimation process is supplied by a rise of the shelf temperature. The ice on the sublimation interface directly changes to water vapor and the vapor then passes the already dried matrix while the sublimation interface moves downwards. The primary drying phase is usually the longest step and therefore shows high optimization potential [7]. To accelerate the sublimation rate of the process, the product temperature should be as high as possible while avoiding collapse and product degradation [8,9]. It is necessary to maintain the product temperature during primary drying under the eutectic melt temperature (for crystalline products) or the collapse temperature (for amorphous products) which is related to the glass transition temperature of the maximally freeze-concentrated solution. The produced product cake should have a short reconstitution time and an elegant appearance [10]. Afterwards, the secondary drying is used to adjust the residual moisture of the product cake [11]. Bound water inside the solute matrix is removed by desorption. To accelerate this process, the shelf temperature is increased drastically. All ice has to be removed beforehand to avoid collapse. Furthermore, the glass transition temperature is a function of the bound water concentration and is a constraint in secondary drying [4].

The product temperature during primary and secondary drying is a function of shelf temperature and chamber pressure [12]. Lyophilization processes have to be developed in a way that ensures product safety while maximizing the productivity.

**Figure 1:**
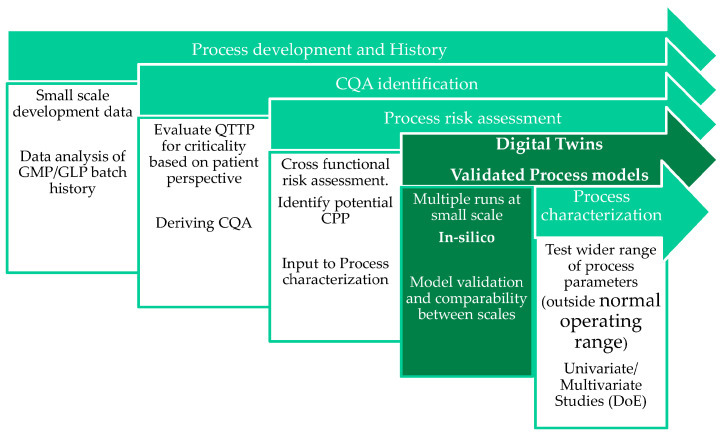
Models as part of process development [21].

Thermocouples, Resistance Thermal Detectors (RTD) or wireless temperature measurement systems can be used to indicate the product temperature. Large amounts of experiments would be necessary to develop and optimize a freeze-drying process based on this data. Here, in early-stage process development, modeling can be used to reduce the experimental workload. Modeling provides a reliable and predictive indication of process behavior based on material properties, models and process parameters. In the Quality by Design approach, demanded by regulatory authorities, process modeling can be used for process-knowledge deepening, design-space exploration and data-driven process optimization (s. Figure 1) [13].

Fully dynamic models, also called sorption-sublimation models, can be used for both primary and secondary drying [14,15,16,17]. Here, the product inside the vial is separated into two regions, a frozen and a dried region, separated by the moving sublimation front at which the ice sublimates. They are able to model the product temperature, water and ice content at different height levels inside the vial for the different regions and can further be extended to model the product in multidimensional space. This exact description of the product dynamics during the drying steps needs complex mathematical equations and time-consuming solution approaches. Therefore, an approach has been developed that takes pseudo-stationary conditions into account during primary drying [2,12,18,19,20]. An energy balance for the frozen region and a mass balance for the ice are formulated. The assumption of pseudo-stationary conditions is reasonable because of the slow dynamics of the process. These model equations are far simpler and therefore the solution is faster at the cost that only the temperatures at the product bottom and on the sublimation interface during primary drying are known.

**Figure 2:**
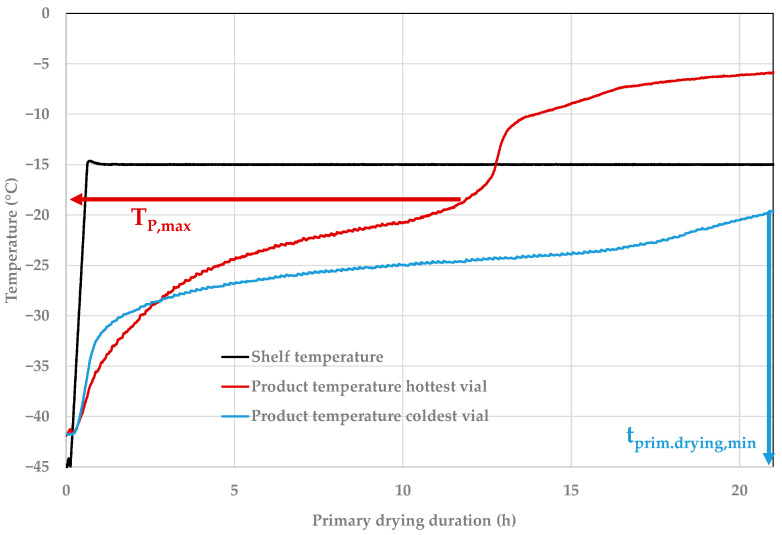
Exemplified product temperature profiles for the hottest and coldest vial [21].

One key challenge in the development of lyophilization processes is the so-called edge effect shown in Figure 2 [22,23]. During primary drying, vials on the edge of the shelf receive a higher radiative amount of heat that alters the drying rate compared to center vials. An unfavorable choice of process parameters can lead to very heterogeneous drying and long processing times. Edge vials are more prone to collapse; therefore, they limit the process parameters’ shelf temperature and chamber pressure while the colder vials define the shortest primary drying time [17]. A succession to secondary drying before all ice is removed would lead to collapse.

In this work, a modeling approach is outlined that is able to predict the product temperature profiles and endpoints of primary drying phases for a wide variety of process parameters. The model is a further development of Juckers et al. [21]. Here, it is validated for an amorphous system and the model results are extended with the product temperature. The process model has been adopted from Velardi et al. [20]. The necessary model parameters are determined by the procedures of Wegiel et al. [24] and Tang et al. [25]. For model validation, the workflow of Sixt et al. has been used [26]. The pseudo-stationary model is validated with an experimental Design of Experiments on a pilot scale freeze-dryer. The simulated results for the endpoint and product temperature profiles are in good agreement for the center vials. The forecasting accuracy increases with more aggressive cycles for the edge vials.

## 2. Materials and Methods

### 2.1. Product Mixture and Instruments

Saccharose (d(+)-saccharose (VWR International, Leuven, Belgium)) is dissolved in purified water (arium™pro, Sartorius AG, Göttingen, Germany) to yield a 25 g/L solution. To measure the weights, a laboratory-scale LC 1200 S (Sartorius AG, Göttingen, Germany) is used.

### 2.2. Freeze-Drying Equipment

The freeze-drying experiments are conducted in a pilot scale freeze-dryer (Epsilon 2-6D LSCplus (Martin Christ Gefriertrocknungsanlagen GmbH, Osterode am Harz, Germany)). 6R vials are used as containers for freeze-drying. The fill volume depends on the experimental point as depicted by the Design of Experiments (DoE). An Eppendorf Research plus a 0.5–5 mL pipette (Eppendorf AG, Hamburg, Germany) is used to fill a total of 135 vials before loading onto the middle shelf. During the experiments, one of the three available shelves is fully loaded with vials. The product temperature during all phases of freeze-drying is measured by Wireless Temperature Measurement plus (WTMplus) sensors.

### 2.3. Experimental Runs

The freeze-drying cycle was adopted from literature [27]. At first, the shelf temperature is lowered to −45 °C and held for 2 h. Afterwards, it is raised to −20 °C and held for 1 h before returning to −45 °C and a final hold time of 2 h. During the freezing and annealing, all temperature ramps are 1 °C/min. The primary drying conditions are varied based on the DoE, shown in Table 1. Here, the final shelf temperature, chamber pressure, fill volume and temperature ramp are varied, and − means that the low value is established, while + stands for the highest value. The mean value is used in the center point (CP). This experimental point is conducted three times for statistical evaluation. The end of primary drying is determined by comparative pressure measurement. As a forwarding condition, a value of 15% is used.

### 2.4. Modeling

In this study, a pseudo-stationary model of the coupled heat and mass transfer is used to calculate the endpoint and product temperature of distinct vials during the primary drying phase. An exact derivation can be found elsewhere [20]. This modeling approach has already been validated for the endpoint determination of a crystalline excipient [21]. The model assumes pseudo-stationary conditions during primary drying. All heat supplied by the heat source, which in this case is the shelf, is used for sublimation. Heat accumulation in frozen and dried zones is neglected. Therefore, the coupled heat and mass transfer can be written as:

(1) $$K_{v}\cdot \left(T_{S}-T_{P}\right)\cdot A_{v}=\frac{\Delta H_{subl}}{R_{P}}\left(p_{i}-p_{c}\right)\cdot A_{p}$$

*R_p_* is the dry layer resistance, *p_i_* is the partial vapor pressure on the sublimation interface, *p_c_* is the chamber pressure and *A_p_* the inner cross-sectional area of the vial. The partial vapor pressure is calculated by inserting the sublimation interface temperature into the new sublimation pressure equation. The temperature on the sublimation interface is calculated through heat conduction:

(2) $$K_{v}\cdot \left(T_{S}-T_{P}\right)={\left(\frac{1}{K_{v}}+\frac{L_{frozen}}{\lambda_{frozen}}\right)}^{-1}\left(T_{S}-T_{i}\right)$$

*L_frozen_* is the length of the frozen area, $\lambda_{frozen}$ the heat conductivity of ice and *T_i_* the sublimation interface temperature. *K_v_* and *R_P_* are model parameters and need to be determined. The exact determination is described as follows.

### 2.5. Overall Vial Heat Transfer Coefficient K_v_

In this study, the gravimetric method is used to obtain *K_v_* for individual vial positions. Additionally, 135 6R vials are filled with water, a selection is weighed, and all vials are loaded onto the middle shelf. The vials are frozen overnight for about 12 h and afterwards the primary drying starts. The shelf temperature and chamber pressure are varied based on the DoE. In all experiments, the temperature ramp to the final shelf temperature is 1 °C/min. Primary drying is aborted after around 4 h and the previously selected vials are weighed again. *K_v_* is calculated by following equation:

(3) $$K_{v}=\frac{\left(\Delta m\cdot \Delta H_{s}\right)/\Delta t}{A_{v}\cdot \left(T_{S}-T_{p}\right)}$$

Δ*m* describes the measured mass difference, Δ*H_s_* is the sublimation enthalpy, Δ*t* is the primary drying duration, *A_v_* the outer cross-sectional area of the vial and *T_S_* and *T_P_* are the shelf and product temperature, respectively. All these parameters are known after the experiment and can be used to calculate the vial heat transfer.

### 2.6. Dry Layer Resistance R_P_

The dry layer resistance is determined by Manometric Temperature Measurement (MTM). An optimized periodic MTM is used every 10 min and the duration varies up to 30 s depending on the pressure increase. The MTM is finished as soon as no significant pressure increase is detected for 3 s. This procedure ensures that the drying process is disturbed for the shortest time possible to obtain the pressure rise data. MTMplus Analyse software (Martin Christ Gefriertrocknungsanlagen GmbH, Osterode, Germany) is used to analyze the data. A Levenberg-Marquardt algorithm fits the MTM equation to the pressure-rise data.

### 2.7. Software

JMP (JMP Inc., SAS Institute, Cary, NC, USA) was used to generate the DoE and to create the pareto chart of standardized effects. During freeze-drying runs, LPCplus collects all data and the MTMplus Analyse software is used for analysis of the pressure-rise data.

## 3. Results

In this section, the experimental and modeling results are shown. First, the results for the model parameter determination are shown. The endpoints and product temperature profiles for the primary drying are simulated with the aid of Monte-Carlo simulations and then compared to the experimental findings.

### 3.1. Vial Heat Transfer Coefficient

**Figure 3:**
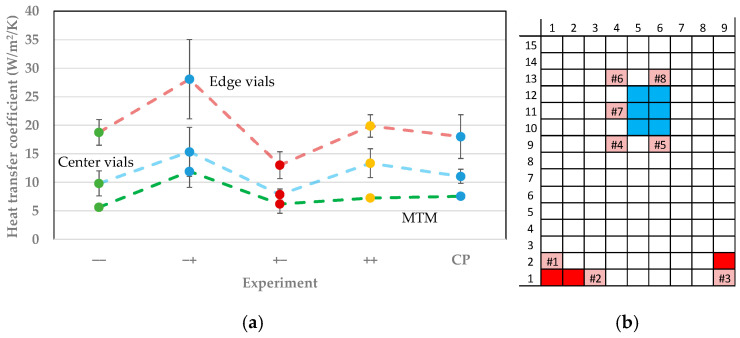
(**a**) Vial heat transfer coefficients for edge (red dotted line) and center (blue dotted line) vials and MTM (green dotted line) for different process conditions. (**b**) Vial and WTMplus position (pink with corresponding number) and vial category (red: edge vial; blue: center vial).

The vial heat transfer coefficient *K_v_* describes the effective heat transfer from the shelf into the vial and are determined by ice sublimation tests. *K_v_* is determined for a selection of vials and by MTM for various process parameters (shown in Table 2). The shelf temperature and chamber pressure have been varied according to the DoE (s. Table 1). The position of the vials and their category is shown in Figure 3b. The product temperature of the nearest WTMplus sensor has been used for calculations.

The results of the heat transfer coefficient are shown in Figure 3a. The vials are grouped into edge and center vials for a clearer display of the results. The error is shown for a 95% confidence interval. The heat transfer coefficient of the edge and center vials have been pooled for an easier presentation. The selected vials and the position of the WTMplus sensors are shown in Figure 3b.

The *K_v_* value for the edge vials is higher throughout the investigated process parameters. During primary drying, these vials receive a higher radiative heat contribution. This explains the edge effect and the resulting faster drying of the edge vials. Altering of process parameters can reduce the edge effect and homogenize the drying. An increase in shelf temperature leads to a significant decrease of *K_v_* of the edge vials from 18.7 to 13 W/m^2^/K. The decrease of the coefficient of the center vials is smaller, from 9.8 to 7.9 W/m^2^/K. The chamber pressure additionally influences the heat transfer. An increased chamber pressure at constant shelf temperature increases *K_v_* because of an improved gas conduction regardless of vial position and shelf temperature. During the experiments, *K_v_* was additionally determined by MTM. The values for *K_v_* are smaller than the experimentally determined values. Since MTM is a batch method based on the pressure-rise test, this method can only yield values for the slowest drying vials. The deviation from the center vial value can either be explained by a general underestimation of the value by MTM or by the fact that the coldest vial was not identified with the selection. Independent of the cause, the estimated value for *K_v_* of MTM can be useful to determine a safe primary drying duration for a development and production environment since the measurement happens simultaneously to the experiment and no additional experimental effort is necessary.

### 3.2. Dry Layer Resistance

The dry layer resistance *R_p_* describes the resistance of the dried matrix to the vapor flow. During the primary drying, the frozen zone recedes due to sublimation, and the height of the dry layer increases. The increase in the dry layer resistance with a growing dry layer is usually described by the following equation:

(4) $$R_{p}=R_{1}+\frac{R_{2}\cdot L_{dry}}{1+R_{3}\cdot L_{dry}}$$

During primary drying MTM is regularly performed to yield the value of dry layer resistance. The parameters of equation four are determined by minimization of the sum of square errors for all experiments, and their values are shown in Table 3.

The variation for the parameters is high because in this study the nucleation of the vial has not been controlled during the freezing step. The different nucleation temperatures in the experiments lead to the high variation [28]. It has further been reported that a change in process parameters alters the dry layer resistance [29].

### 3.3. Endpoint Determination

The endpoint of primary drying is a critical process parameter. All ice must be removed by sublimation before secondary drying begins to prevent collapse. However, a primary drying time well above complete ice removal lowers productivity.

**Figure 4:**
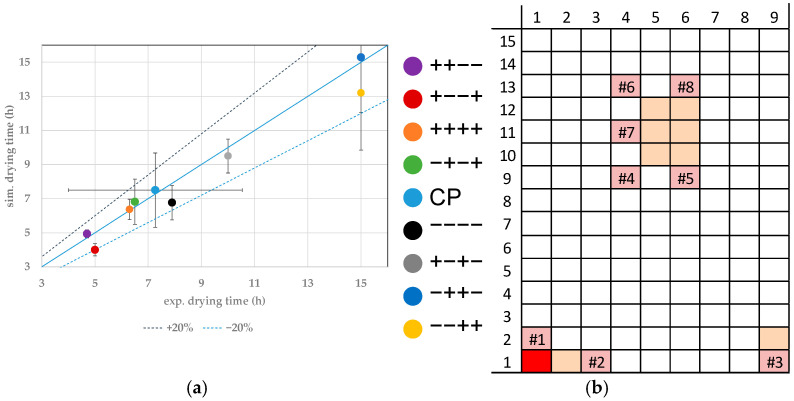
(**a**) Parity plot between simulated and experimental primary drying endpoints for vial 1.1. (**b**) Color legend for experiments and vial position (red: shown vial, orange: other probed vials, pink: WTMplus sensor position and WTMplus number).

In this study, the experimental endpoints for individual vials are determined through WTMplus sensors. A sharp increase in product temperature detects the endpoint. In the simulations, the ice content is simulated, and a complete removal determines the endpoint. For the comparison of experimental and simulated primary drying endpoints, the result of the closest WTMplus sensor is used. Both endpoints are compared with parity-plots. At first, the results for edge vials are shown. In Figure 4a, the parity plot for the vial 1.1 is shown. A Monte Carlo simulation has quantified the simulated endpoint error for all vials. Here, the model parameters are varied inside their experimentally determined boundaries. The center point has been conducted three times and the error is calculated through reproducibility. Both errors show a confidence of 95%.

For vial 1.1 measured with WTMplus#1, the fastest drying has been achieved at the experimental point ++−−. High shelf temperature and chamber pressure lead to an increased heat transfer into the product. Combined with a low filling volume, this results in the fastest drying of 4.7 h, while the simulations calculate a value of 4.94 h. The second fastest drying for vial 1.1 has been achieved at +−−+. Here, the shelf temperature and temperature ramp are high, while chamber pressure and fill volume are set to the low value. The lower chamber pressure leads to a decreased heat transfer into the product compared to the fastest drying point. This is nearly compensated by the high temperature ramp to the final shelf temperature. The vial reaches the temperature at which the ice vapor pressure exceeds the chamber pressure faster and sublimation therefore occurs earlier, resulting in a comparable drying time. The experimental endpoint has been determined at 5 h while the simulation results an endpoint at 4 h. Here again, simulation and experiment show good agreement.

The slowest primary drying phase is found in the experiments −++− and −−++. The experimental drying time has been determined at 15 h for both experiments. In both experiments, the shelf temperature is set to the lowest value of the DoE and a high fill volume. Low heat transfer paired with an increased fill height leads to longer process duration. Here again, as for the fastest drying experiments, an increase in temperature ramp compensates the lower heat transfer of the decreased chamber pressure. All experimental endpoints show good agreement with the simulations.

**Figure 5:**
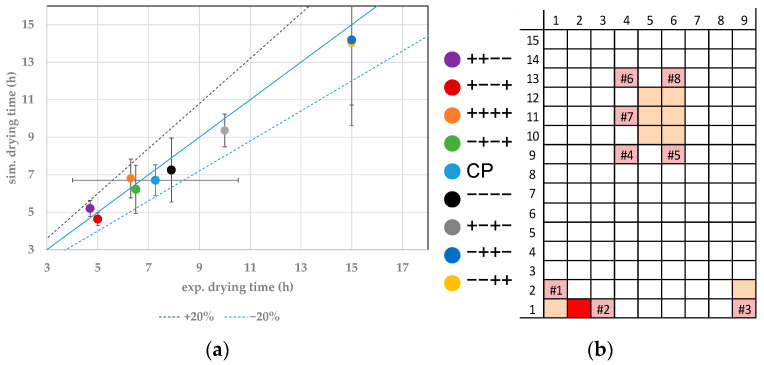
(**a**) Parity plot between simulated and experimental primary drying endpoints for vial 1.2. (**b**) Color legend for experiments and vial position (red: shown vial, orange: other probed vials, pink: WTMplus sensor position and WTMplus number).

For edge vial 1.2, the endpoints are compared in Figure 5. Here, the same results can be extracted as from vial 1.1. It should particularly be emphasized that the test ++++ shows a significantly faster primary drying than the test −−−−, although the filling volume was doubled. The right choice of process parameters is detrimental for a time efficient and safe product.

**Figure 6:**
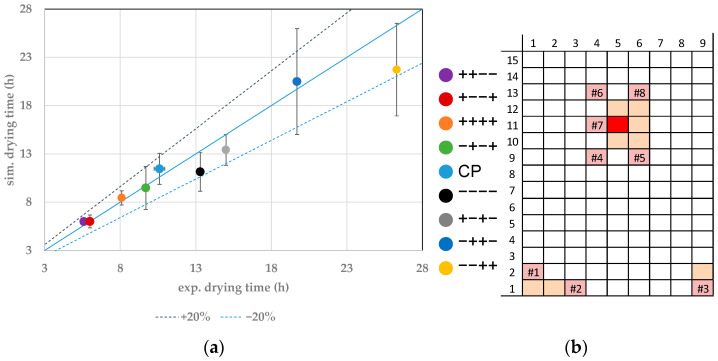
(**a**) Parity plot between simulated and experimental primary drying endpoints for vial 11.5. (**b**) Color legend for experiments and vial position (red: shown vial, orange: other probed vials, pink: WTMplus sensor position and WTMplus number).

The comparison of experimental and simulated endpoints has also been made for a selection of center vials. Here, vials 11.5 and 12.6 are shown. Figure 6 shows the parity plot for vial 11.5. This vial is one of the slowest drying vials studied. The fastest drying is achieved in the experiments ++−− and +−−+, with 5.6 h and 6 h, respectively, and the simulations resulted in a drying endpoint of 5.9 h and 6 h, respectively. Compared to the edge vial 1.1, the drying takes 1 h longer and therefore results in a relative difference of 16.7%. The slowest drying can be determined in the experiment −−++. The experimental and simulated endpoints lay at 26 h and 21.7 h, respectively. Again, this result is compared to the drying process of vial 1.1. A difference of 11 h (relative 42%) for the experiments can be determined. The simulated endpoint shows a difference of 8.5 h (relative 32%). The modeling approach therefore can also quantify the edge effect of the studied vials.

**Figure 7:**
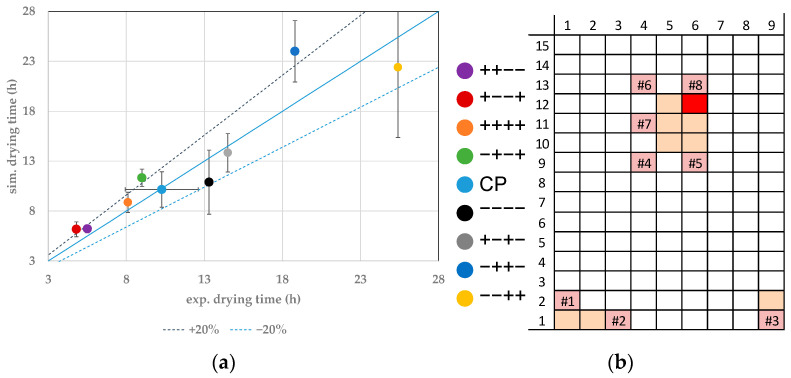
(**a**) Parity plot between simulated and experimental primary drying endpoints for vial 12.6. (**b**) Color legend for experiments and vial position (red: shown vial, orange: other probed vials, pink: WTMplus sensor position and WTMplus number).

Figure 7 shows the results for vial 12.6. The experimental and simulated primary drying endpoints show sufficient agreement.

**Figure 8:**
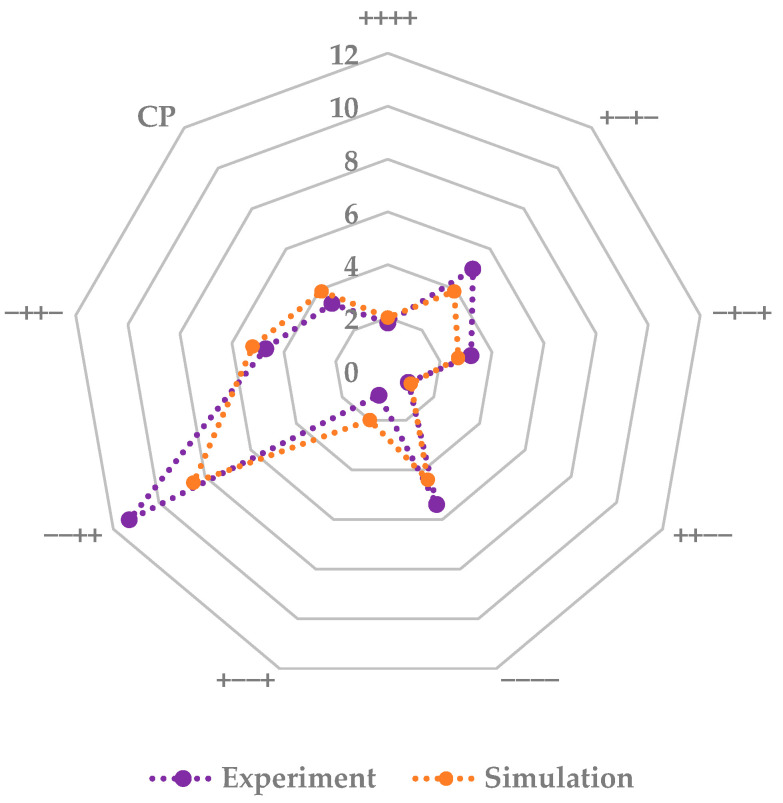
Quantification of edge effect for primary drying time difference.

The choice of process parameters not only influences the drying time for each vial, but it also influences the edge effect. This influence is shown in Figure 8. The drying behavior of the experiments with the lowest shelf temperature and chamber pressure show the highest heterogeneity. For experiment −−−−, the experimental difference is 5.4 h, and for the simulation it is 4.4 h. Increasing fill volume leads to a higher heterogeneity of 11.3 h in experiments or 8.5 h in simulations. Optimizing the process parameters decreases the batch heterogeneity. An increase in shelf temperature and/or an increase in chamber pressure decreases the batch heterogeneity to a value of 0.9 h(experiments)/1 h(simulations) in the experiment ++−−. An aggressive cycle design provides more uniform drying of the batch.

**Figure 9:**
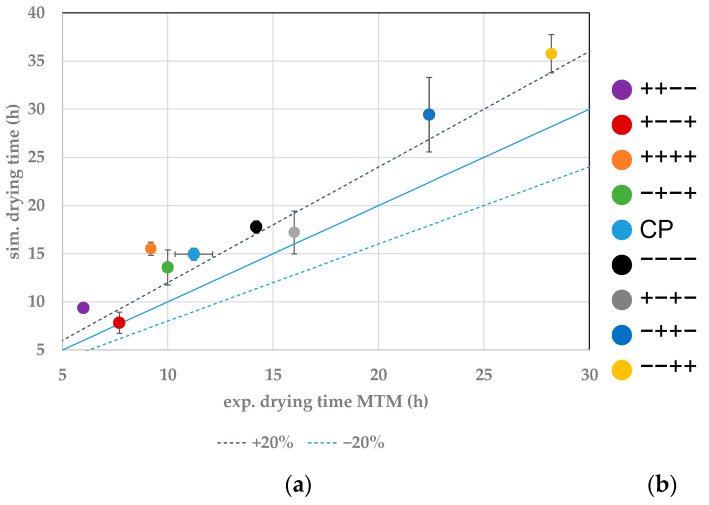
(**a**) Parity plot between simulated and experimental primary drying endpoints of MTM. (**b**) Color legend.

Additionally, to the endpoints of the distinct vial, the batch endpoint has been calculated with the help of the model parameter yielded solely by MTM. Figure 9 shows the results. In the parity plot, the simulated primary drying endpoints are compared to the endpoint determined by MTM. This endpoint is reached as soon as the vapor pressure of ice is equal to the chamber pressure. As can be seen, the simulated results overestimate the primary drying endpoint in most experiments. This is mainly caused by the lower value of the heat transfer coefficient. An optimization solely based on this model parameter would most likely end in the collapse of some vials because the product temperature in the simulation is underestimated. Nevertheless, the results obtained by MTM are useful for designing a safe primary drying time after optimization with distinct model parameters has been utilized.

**Figure 10:**
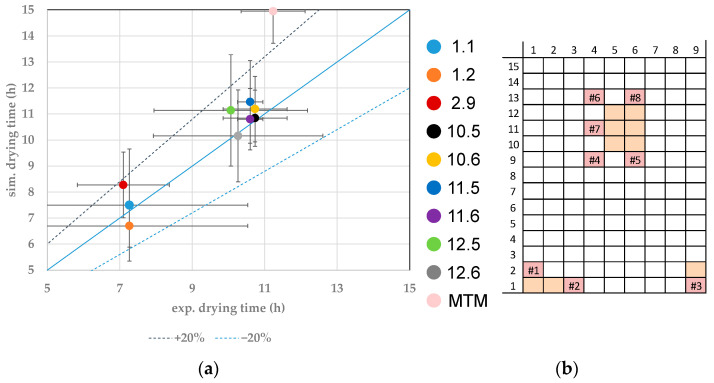
(**a**) Parity plot between simulated and experimental primary drying endpoints for distinct vials at the CP. (**b**) Color legend and vial position (orange: probed vials, pink: WTMplus sensor position and WTMplus number).

To evaluate the accuracy and precision of the model for the prediction of the primary drying endpoint, the center point has been calculated three times to allow statistical evaluation. The results are shown in Figure 10. The edge and center vials can be clearly identified. Edge vials dry faster than center vials, and small deviations in drying behavior inside the respective category can be detected.

Accuracy describes the correct prediction of experimental data within the parameter set. Experimental and mean value show sufficient agreement for the vials which model parameters have been distinctly determined. Precision describes the effect of the uncertainties of the model parameter on the simulated results. Since the simulation shows smaller deviations than the reproducibility of the experimental data, the precision of the model is sufficient.

**Figure 11:**
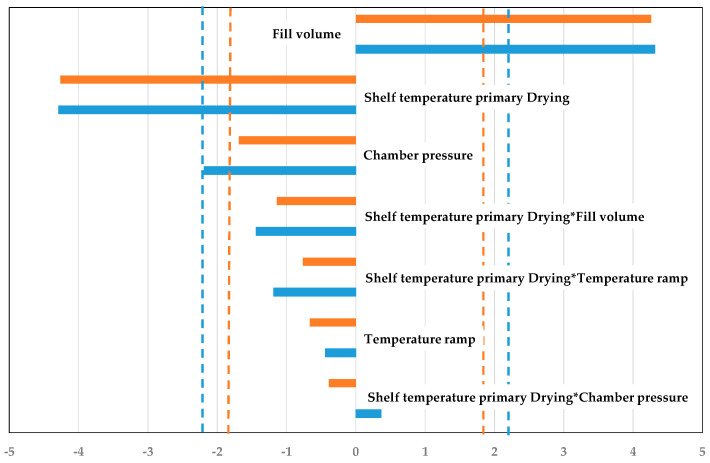
Pareto diagram of standardized effects for primary drying duration (blue: Experiment; orange: Simulation, dashed line: significance threshold).

To access the interaction and strength of different process parameters for simulation and experiment, pareto plots are used. Pareto plots define statistically relevant process parameters on the performance and their direction. They are shown in Figure 11. Both the experiment and the simulation show the same significant parameters, fill volume and shelf temperature. Fill volume correlates positively with the primary drying duration, while an increase in shelf temperature leads to a reduction. This is consistent with the previously elaborated statements. ++−− and +−−+ show the fastest drying, while −−++ and −++− have the slowest drying.

The simulation shows higher precision and accuracy as described above and is able to describe the parameter interactions and strengths, just as the experiments do. Therefore, this modeling approach can be used to substitute experiments for process development and optimization of the primary drying endpoint.

### 3.4. Product Temperature Profile

The knowledge of the product temperature profile is of utmost importance for process development, optimization and control. During freeze-drying, the product temperature must not exceed the critical temperature of the formulation to obtain an elegant product.

**Figure 12:**
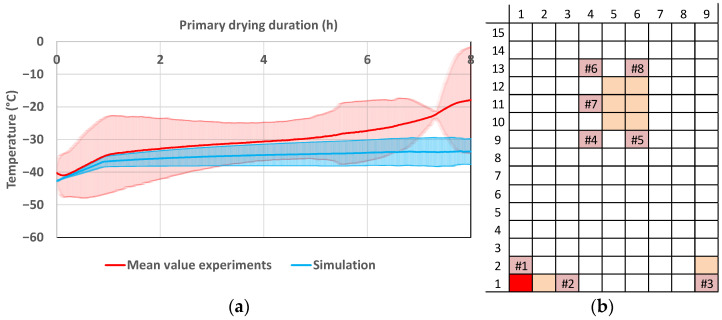
(**a**) Product temperature profile of vial 1.1 during primary drying at the CP. (**b**) Vial position (red: shown vial, orange: other probed vials, pink: WTMplus sensor position and WTMplus number).

In Figure 12, the product temperature profiles of vial 1.1 can be seen. The experiments are shown in red and the simulations in blue. In the beginning, simulated and experimental data are in good agreement, but with ongoing primary drying the data drift apart. Vial 1.1 is an edge vial and the additional radiative heat transport leads to big deviations in the product temperature. For the edge vials, the modelling approach shows a good precision, but the accuracy is lacking. At around 7.5 h, the mean value of the primary drying endpoint is reached; therefore, the experimental temperature shows a smaller deviation.

The assumption of steady-state conditions for the heat transfer is lacking for edge vials where the radiative input is high. Not all heat supplied is used for sublimation. Heat accumulation inside the frozen layer is not negligible and also effects the product temperature. Since the model does not take this phenomenon into account, the product temperature is underestimated. This has to be taken into account for process optimization to eliminate the risk of the collapse of edge vials.

**Figure 13:**
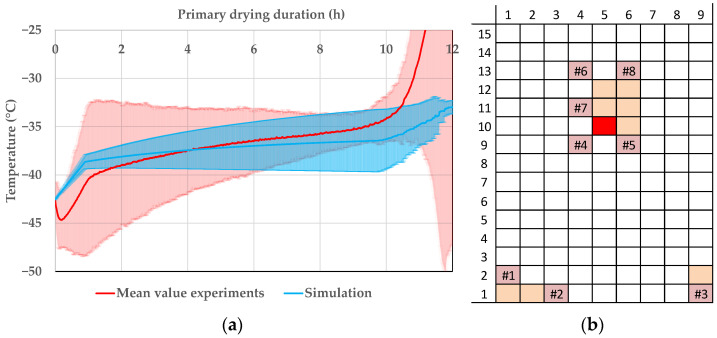
(**a**) Product temperature profile of vial 10.5 during primary drying at the CP. (**b**) Vial position (red: shown vial, orange: other probed vials, pink: WTMplus sensor position and WTMplus number).

**Figure 14:**
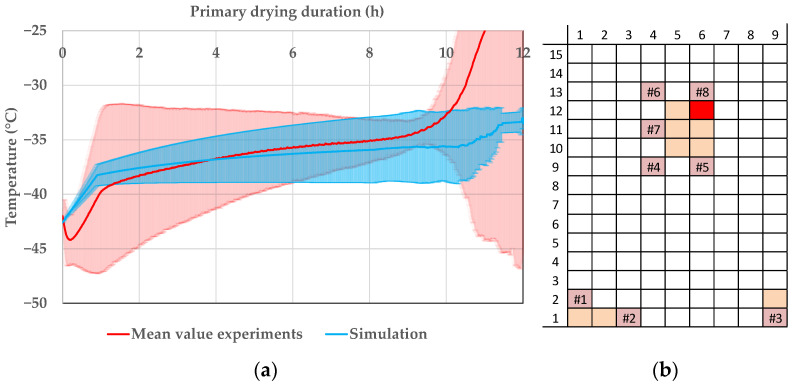
(**a**) Product temperature profile of vial 12.6 during primary drying at the CP. (**b**) Vial position (red: shown vial, orange: other probed vials, pink: WTMplus sensor position and WTMplus number).

The situation is different for the center vials. As examples, vial 10.5 and 12.6 are shown in Figure 13 and in Figure 14, respectively. The simulated and experimental product temperature profiles are in good agreement. The simulation is able to accurately determine the product temperature profile of the vials 10.5 and 12.6. It shows sufficient accuracy. Since the simulation is able to predict the maximum temperature of the experiments, the precision is additionally sufficient for process development and optimization.

The accuracy for the prediction of the edge vial is lacking since the experimental point has one of the highest deviations of edge vials to center vials (s. Figure 8). The high proportion of radiant energy means that in these vials, the assumptions of the model are conditionally valid. Here, pseudo-stationary conditions for sublimation do not apply as in the center vials, which can be described with sufficient accuracy and precision.

**Figure 15:**
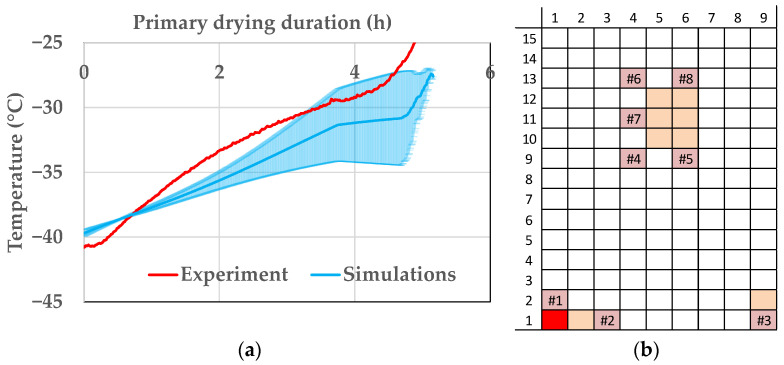
(**a**) Product temperature profile of vial 1.1 during primary drying at ++−−. (**b**) Vial position (red: shown vial, orange: other probed vials, pink: WTMplus sensor position and WTMplus number).

However, as already stated in the endpoint-determination section, the edge effect depends on process conditions. The product temperature profiles for the experiment with the lowest deviation between edge and center vials are compared. Figure 15 shows the comparison of the simulation results with the experiment. The product temperature data are in good comparison and the experiment lays within the simulation deviation caused by uncertainty of model parameters at the end of primary drying, where the risk of exceeding the critical temperature is highest. The model shows satisfactory accuracy for these conditions and is able to calculate the product temperature profile of an edge vial if the edge effect is lowered by an adequate selection of process parameters.

**Figure 16:**
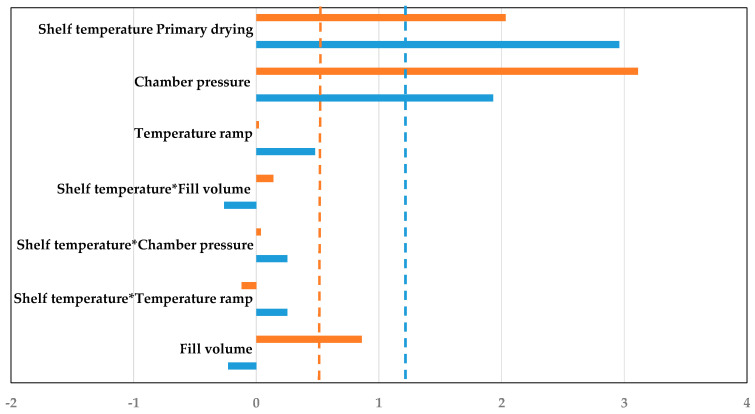
Pareto diagram of standardized effects for primary drying product temperature (blue: Experiment; orange: Simulation, dashed line: significance threshold).

Lastly, as in the endpoint determination part, pareto plots are used to access the strength and interaction of the different process parameters (s. Figure 16). Shelf temperature and chamber pressure are the significant parameters for experiment and simulation. An increase of the respective value increases the supplied heat to the vial. Only in the simulation is the fill volume another significant parameter. An increased fill volume leads to a higher dry layer, which results in an increased dry layer resistance which finally causes a higher product temperature. Here, the sensitivity of the modelling approach has to be optimized in order to describe the experiments more accurately.

The simulation shows higher precision and accuracy for the center vials and is able to describe the parameter interactions and strengths, just as the experiments do. The prediction efficiency increases for edge vials with more aggressive drying cycles. Therefore, this modeling approach can substitute experiments and aid in the exploration of the design space.

## 4. Discussion

This paper proposes a distinct method for endpoint determination and product temperature prediction by a modeling approach based on Velardi et al. [20] and combined by the distinct model parameter determination of Wegiel et al. [24] and Tang et al. [25]. Sucrose has been used as an exemplary material system because it is a popular excipient for freeze-drying. A pseudo-stationary process model has been developed. The required model parameters *K_v_* and *R_p_* are distinctly determined with a reproducible model-parameter determination concept. *K_v_* is determined by ice sublimation tests while *R_p_* is determined via MTM. Both model parameters show the expected physical behavior.

The primary drying step in lyophilization shows high optimizing potential because it is usually the longest process step. The determination of the primary drying endpoint and the product temperature profile are of utmost importance to design a safe and productive primary drying cycle. An experimental fractional factorial DoE has been used for experiments in which all parameters for the primary drying process (shelf temperature, chamber pressure, temperature ramp and fill volume) are varied.

To validate the model for the endpoint determination, the simulated results are compared to the experimental findings. It has been shown that the model can accurately predict the endpoint of primary drying and the deviations established by Monte Carlo simulations are smaller than the experimental. Thus, the model shows sufficient accuracy and precision, and is therefore validated for the endpoint determination. Additionally, the model could accurately determine the influence of process parameters on the edge effect and their significance on the drying through pareto plots. A more aggressive cycle leads to an increase in drying uniformity of the batch.

The model has further been validated for the product temperature profiles. Therefore, experimental and simulated results have been compared again. The product temperature profiles of center vials can be accurately predicted. The prediction of the profile of edge vials is more sophisticated since their drying behavior alters due to the edge effect. However, with suitable process parameters, the edge effect can be reduced and the prediction accuracy of edge vials increases. Therefore, the process model is validated for the product temperature profile prediction.

The modeling approach is able to calculate the primary drying endpoint and product temperature profile of distinct vials. It can be used for accelerated process design and optimization in early process development to reduce the experimental workload.

The presented, data-driven approach for product temperature and endpoint determination has been proven to be successful. A more profound insight into CPP and their interaction could be achieved. This approach will now be tested for the design and optimization of primary drying and will be further developed into a sorption-sublimation model in order to be able to describe the experiments even more precisely with the aid of the higher modeling depth.

## Figures and Tables

**Table 1:** Experimental Design of Experiments (DoE).

#		Primary Drying			
		Shelf Temperature (°C)	Chamber Pressure (mbar)	Fill Volume (mL)	Temperature Ramp (°C/min)
1	++++	0	0.15	2	1
2	+−+−	0	0.05	2	0.2
3	−+−+	−25	0.15	1	1
4	++−−	0	0.15	1	0.2
5	−−−−	−25	0.05	1	0.2
6	+−−+	0	0.05	1	1
7	−−++	−25	0.05	2	1
8	−++−	−25	0.15	2	0.2
9	CP	−12.5	0.1	1.5	0.6
10	CP	−12.5	0.1	1.5	0.6
11	CP	−12.5	0.1	1.5	0.6

**Table 2:** Process parameters for *K_v_* determination.

		Shelf Temperature (°C)	Chamber Pressure (mbar)
1	++	0	0.15
2	+−	0	0.05
3	−+	−25	0.15
4	−−	−25	0.05
5	CP	−12.5	0.1

**Table 3:** Dry layer resistance parameters.

Parameter	Value
*R*_1_ (m/s)	26,834 ± 7404
*R*_2_ (1/s)	1.62 × 10^7^ ± 1.9 × 10^7^
*R*_3_ (1/m)	42.76 ± 181.47
